# Obituary: Professor Dr Javier Abadía, 1954–2022

**DOI:** 10.3390/plants12183245

**Published:** 2023-09-13

**Authors:** Jorge Rodríguez-Celma, Ana Álvarez-Fernández

**Affiliations:** Plant Stress Physiology Group, Plant Biology Department, Estación Experimental de Aula Dei, Consejo Superior de Investigaciones Científicas (EEAD-CSIC), Avenida de Montañana 1005, E-50059 Zaragoza, Spain; j.rodriguez.celma@csic.es

The unexpected death of Javier Abadía, Research Professor of the Spanish National Research Council (CSIC), deeply shocked all who knew him. Born in 1954 in Zaragoza (Aragón, Spain), Javier was an internationally known expert on plant nutrition. He died at the age of 68, on 18 November 2022, while enjoying hiking—his favourite hobby—with three of his best friends on the trial of the Valdoria ravine in Albalate del Arzobispo (Teruel, Aragón, Spain).

Javier worked for 41 years at the Aula Dei Experimental Station (EEAD), an institute located in Zaragoza which houses the CSIC’s agricultural research in Aragón (Spain). At the EEAD, he led the ‘Plant Stress Physiology’ research group, dedicated to the study of crop responses to different abiotic stresses, with a special interest in iron deficiency. In love with science and his family, he combined both aspects throughout his professional career, and it was common for his family, María Pilar and Nacho, to attend scientific conferences. Often, the relationships with his colleagues transcended work, becoming personal friendships. The numerous condolence messages received from national and international universities and research centres just hours after the sad news of his death was released show the collaborative nature of his scientific career. His colleagues (most of them also friends) equally highlight his scientific and human value. A tribute ceremony organized by his group, the EEAD, and the CSIC was held in Zaragoza shortly after Javier’s passing. The number of attendants (close to 150), personal testimonies (20 direct speeches of doctoral students, disciples, and national and international collaborators) and written messages (more than 100 from all over the world) give testimony of Javier´s legacy in all sectors of the scientific community. Some of those words dedicated to him are at the end of the text of this obituary.

Javier was educated in science since childhood. His father, Armando Abadía, was a prominent chemist whose scientific research was devoted to soil and plant nutrition at the EEAD-CSIC. Following his steps, Javier also graduated in Chemistry at the University of Zaragoza and pursued his PhD at the same institution, the EEAD-CSIC (1978–1984). In 1988, Javier Abadía, after completing two postdoctoral stays at the Universities of California in Berkeley (USA, 1984–1985) and Essex (United Kingdom, 1987), created, together with his sister, Anunciación Abadía, also a Doctor in Chemistry, the ‘Plant Stress Physiology’ group at EEAD-CSIC. He dedicated his entire long career (1978–2022) to the study of the physiological and biochemical mechanisms that higher plants develop in response to environmental stress, including both metal deficiencies and toxicities and other abiotic stresses. His early studies tackled iron chlorosis, a worldwide spread nutritional disorder, endemic to some relevant crops (e.g., fruit crops), especially when grown in calcareous soils such as peach trees in his home region, Aragón. This line of work was the focus of his career, as is reflected by the image of chlorotic peach leaves in the logo of his research group. He became a reference scientist in this area of plant nutrition, publishing 124 SCI articles on the subject. From 1985 to 2000, he significantly contributed to revealing the photosynthetic characteristics of iron chlorotic leaves, studying changes in chlorophyll fluorescence, photosynthetic pigments, and mineral composition in sugar beet and fruit crops. He also revealed a boost in carbon fixation in roots and its transport to the leaves to maintain basic metabolic processes in carbon-starved foliage. His team’s studies on iron-deficient roots also brought the first report of flavin sulfates. In that time period, his research also addressed the effects of salinity stress, water deficit, and high light and temperature on photosynthetic efficiency and mineral nutrition in Mediterranean plants such as barley and oak. In the following decades, he was particularly prolific, publishing an average of six SCI articles per year. From 2001 to 2010, his work on iron deficiency in plants provided evidence on the reliability of floral analysis as a tool for diagnosis, on the effectiveness of foliar fertilization as a correction method, on the root-to-shoots transport of complexes of iron-citrate, on the root responses of several tree species (e.g., cork oak, kiwifruit, orange and Prunus rootstocks), and on the co-ordinated downregulation of light absorption, photosystem II, and Rubisco carboxylation efficiencies in the chlorotic foliage. With his team, he also developed high-resolution mass spectrometry-based methods for highly selective and sensitive determination of plant metabolites mainly relevant to iron homeostasis (e.g., metal complexes), and investigated the effects of toxicities of zinc, cadmium, and lead on photosynthesis and iron acquisition. From 2011 to 2022, he was very interested in the role of root exudates as facilitators of iron uptake: his team achieved the identification of metabolites (e.g., coumarins and flavins) involved in the process and their role in the rhizosphere, and, in collaboration with other laboratories, they identified molecular components involved on the biosynthesis of such compounds and their secretion by roots. Also in collaboration, his team revealed changes in the proteomic profiles of tissues, membranes, and fluids upon metal stresses (e.g., the deficiency and toxicity of iron and manganese). Javier, throughout his career, always remained at the technical forefront, initially using chlorophyll fluorescence, high-performance liquid chromatography, and atomic spectroscopy and later pioneering the application of mass spectrometry techniques in agricultural sciences, including plant metabolomics and proteomics.

The training of new scientists was key to his vision of science. He trained 18 PhDs (3 more in progress) with his team, and a total of 149 researchers from 19 different nationalities have been trained at some point in their careers under his supervision. His team defines its leader as passionate about science and training the new generation of researchers, a tireless worker, very interested in technology, a lover of always doing things right, a fervent practitioner of the peer review system, a promoter of young scientist careers, with great concern to learn about other cultures and their scientists, and eager to provide opportunities for bright students wherever they came from. His team also highlights his desire to communicate results to the scientific community, which is reflected in his written legacy: 195 publications in journals indexed in the ‘Science Citation Index’ database, 40 dissemination communications, 6 book chapters, and the editions of 2 books (ORCID 0000-0001-5470-5901; Scopus ID: 7006255100; Web of Science ResearcherID: H-3123-2019).

Javier was very active in scientific forums, with 241 communications at conferences. His participation was especially relevant in the ‘International Symposium on Iron Nutrition and Interaction in Plants’ (ISINIP), a biennial meeting focused on the study of iron in plants, whose seventh edition he organized in Zaragoza in 1993. In fact, he participated uninterruptedly in the ISINIP from its second edition held in Utah (USA) in 1983 to the most recent held in 2022 in Reims (France), and he was part of its steering committee between 1991 and 2012, of which he was president from 1993 to 1995. He was also an active member of the Spanish Society of Plant Physiology (SEFV, currently SEBP), organizing two conferences for this society: the IX Iberian Symposium on Plant Mineral Nutrition (2002) and the XI Spanish-Portuguese Congress of Plant Physiology (2009).

As a fervent practitioner of “peer review,” Javier contributed with passion and dedication to the evaluation of scientific activity at all levels. He was part of the editorial committees of the journals *Biometals* (2006–2009), *Functional Plant Biology* (2010–2022), and *Frontiers in Plant Nutrition* (2011–2015), and he reviewed articles for up to 37 scientific journals, mostly belonging to the area of ‘Plant Science.’ He acted as an external evaluator for evaluation agencies in Spain, Italy, Israel, and the USA. He was also a member of doctoral thesis committees at the major universities across Spain and some European ones (e.g., Universities of Copenhagen, Bologna and Lund).

Javier was committed not only to his group but also to the development and improvement of both the institute (EEAD) and the organization (CSIC) for which he worked. He was the director of the EEAD from 1994 to 1998 and vice-director between 2002 and 2004. Afterwards, he raised funds and supervised the installation of scientific equipment that is the basis for the creation of current Scientific-Technical Services of the EEAD. At the CSIC, it is worth highlighting his participation as a member of the Agricultural Sciences Area Committee between 2004 and 2008. This committee supervised and guided scientific research in the 13 research institutes that the CSIC had in this area. Javier led the creation of an international commission of experts for evaluating the institutes, and his work was decisive in optimizing and updating their scientific instrumentation. He also participated as a president or member of selection process courts for admission to the scales of Research Professors and Scientific Collaborators (the current scale of Staff Scientists) of the organization.

In addition to being a great mountaineer, he was a great photographer, a hobby inherited from his father. Despite the vast photo library that he leaves us, it is not easy to find him in it. Rather, he photographed others, skilfully avoiding the lens of other cameras, which reflects his little desire for prominence. Even so, among the photos we have obtained, here we include one that reflects another of his great passions, enjoying the gastronomy of any point of the planet he visited. This photograph shows him enjoying a Moorish tea at the restaurant ‘Le Phenicien’ in Philippeville, a town near the Charleroi airport, where he arrived unexpectedly with his team on 2 July 2022 after Ryanair cancelled their flight to Paris on the way to the 20th edition of his favourite congress, the ISINIP. Javier was retiring in less than a year after finishing the writing of some pendent manuscripts and a research project on course. He was teeming with life, thrilled about his next stage, wishing to have more time with his family and friends and for travelling and hiking. His scientific legacy, humankind, and love will remain with all of us who met him forever.

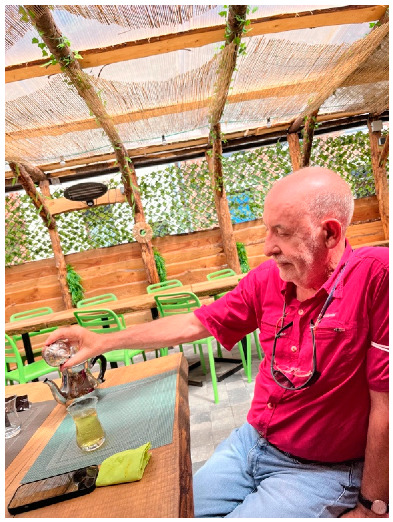



Selection of written messages:

*“I deeply regret that Javier passed away—and so unexpectedly. I will definitely keep Javier in my memory as an excellent and inspiring scientist, and warm-hearted colleague with a great spirit.”*

Nicolaus Von Wiren, Leibniz-Institute of Plant Genetics & Crop Plant Research, Gatersleben, Germany.

*“Abadía J. was the name featured in numerous articles that have always helped and stimulated me in my work. Behind that name was a person of extreme humanity, fairness, respect and humility.”*

Gianpiero Vigani, University of Turin, Turin, Italy.

*“I will remember Javier a real mentor. He was always curious and interested in understanding and discussing new projects and discoveries. At the same time, there was always a smile on Javier’s face. There was no doubt that he enjoyed being in the lab and doing science.”*

François Lefèvre, Université Catholique de Louvain, Louvain, Belgium.

*“I would highlight Javier’s willingness to collaborate in tackling and solving problems. He always dashed to find information and help for planning the appropriate experiments.”*

Jesús Cuartero, Institute of Subtropical and Mediterranean Horticulture “La Mayora”, IHSM-UMA-CSIC, Málaga, Spain.

*“He encouraged me. With his support, I reenergized again and found a third partner and we successfully applied to the project. This was my first real project writing experience and his support meant a lot to me.”*

Seçkin Eroğlu, Middle East Technical University, Ankara, Turkey.

*“When we have someone who marks us, like Javier did and whom we admire so much, we convince ourselves that he will always be around, and we still have a hard time believing that he left us.”*

Maribela Pestana, University of Algarve, Faro, Portugal.

*“I personally appreciated greatly his friendship, great experience and wisdom, as well as his ability to group people around. You could see that his lab was his second family.”*

Stephane Mari, Institute for Plant Sciences of Montpellier, University of Montpellier/CNRS/INRAE/Institute Agro, Montpellier, France.

*“I was always amazed by how Javier was able to say things in a clear, concise and critic manner, but always in a respectful way. Always, spiced up with a half-smile that would necessarily make you feel part of the game.”*

Vicente Pallás, Institute for Plant Molecular and Cellular Biology, IBMPC-UPV-CSIC, Valencia, Spain.

*“However, the most important help I obtained from him was his kind moral support throughout the many years of friendship.”*

Eustaquio Gil, Aula Dei Experimental Station, EEAD-CSIC, Zaragoza, Spain.

*“Maybe it is the good people’s privilege to pass away suddenly.”*

Ádám Solti, Eötvös Loránd University, Budapest, Hungary.

*“I still like to think that he stays there, somewhere, probably in his beloved mountains, and that anytime I will receive a message from him, as we used to interchange in special days.”*

Yolanda Pechero, Gijón Oceonagraphy Center, IEO-CSIC, Gijón, Spain.

*“It has been a real pleasure for me to have the opportunity to speak so long with him during our last ISINIP meeting together in France, we had breakfast together, walked together and I promised him to come and visit him in Spain.”*

Irene Murgia, University of Milan, Milano, Italy.

*“Since I have been under trial times with research and life with a child, the time with Prof. Javier and his team pushed me to go ahead and be brave.”*

Tomoko Nozoye, University of Tokyo, Tokyo, Japan.

